# Giant cell arteritis: reviewing the advancing diagnostics and management

**DOI:** 10.1038/s41433-023-02433-y

**Published:** 2023-02-14

**Authors:** Edward J. Bilton, Susan P. Mollan

**Affiliations:** 1 Ophthalmology Department, Queen Elizabeth Hospital, University Hospitals Birmingham NHS Foundation Trust, Birmingham, B15 2TH UK; 2 INSIGHT Health Data Research hub for eye health, University Hospitals Birmingham NHS Foundation Trust, Birmingham, B15 2TH UK; 3 Transitional Brain Science, Institute of Metabolism and Systems Research, College of Medical and Dental Sciences, University of Birmingham, Birmingham, B15 2TT UK

**Keywords:** Diseases, Eye manifestations, Risk factors, Diagnosis, Prognosis

## Abstract

Giant Cell Arteritis (GCA) is well known to be a critical ischaemic disease that requires immediate medical recognition to initiate treatment and where one in five people still suffer visual loss. The immunopathophysiology has continued to be characterised, and the influencing of ageing in the development of GCA is beginning to be understood. Recent national and international guidelines have supported the directed use of cranial ultrasound to reduce diagnostic delay and improve clinical outcomes. Immediate high dose glucocorticoids remain the standard emergency treatment for GCA, with a number of targeted agents that have been shown in clinical trials to have superior clinical efficacy and steroid sparing effects. The aim of this review was to present the latest advances in GCA that have the potential to influence routine clinical practice.

## Abstract (zh)

众所周知, 巨细胞动脉炎 (GCA) 是一种严重的缺血性疾病, 需要进行迅速的诊断后才能治疗, 但五分之一的患者仍会导致视力损伤。其免疫病理生理学特征正不断地被描述出来, 人们也逐渐意识到衰老对GCA进展的影响。新近的国家与国际指南中都支持直接使用颅脑超声来减少诊断过程中的延误并改善临床结局。快速使用大量糖皮质激素仍然是GCA的标准急救方法, 尽管一些靶向药物在临床实践中已经证实具有更好的临床疗效且具有类固醇的效果。本综述旨在介绍GCA的最新进展, 这可能会对日常的临床实践产生影响。

## Introduction

Giant cell arteritis (GCA) is the most common form of vasculitis with a pooled incidence rate of 10 per 100,000 people over the age of 50 years old [1]. The prevalence in England has been shown to be rising, with increased numbers of people being investigated for suspected GCA and increased recognition of sight loss [2]. World-wide by 2050 over 3 million people will be expected to be diagnosed with GCA and half a million are predicted to have permanent vision loss [3].

Although there has been greater awareness of GCA in recent years, its varied presentation still leads to diagnostic uncertainty amongst healthcare professionals. As such clear diagnostic criteria, accessible specialist referral pathways and informative management guidelines are vital for prompt diagnosis and appropriate initiation of treatment [4–8].

## Epidemiology

The incidence of GCA is higher in the northern hemisphere, with the highest incidence being recorded in Scandinavia of 21.6 per 100,000 people, as compared to the European incidence being 7.3 per 100,000 [1]. Epidemiology publications on the incidence in Olmsted County, USA which have been extrapolated to reflect the incidence in the USA, may have been an overestimate as the County have a higher portion of people with Scandinavian ancestry [1, 9]. Therefore, the geographical distribution is as expected strongly linked with genetic susceptibility [10–12]. GCA has been reliably associated with major histocompatibility complex molecules (i.e. HLA-DR3, HLA-DR4, HLA-DR5 and HLA-DRB1) particularly with carriage of HLA-DRB1*04 alleles [13]. GCA predominantly affects people ≥50 years of age, with rising prevalence in the context of an aging population and peak in the 7th decade [14]. Women are two and half times more likely to acquire the condition than men [1, 15].

## Pathophysiology

Characterised by granulomatous infiltration, GCA is a product of inappropriate T cell migration and subsequent inflammatory cytokine release into the vascular adventitia. In the simplest terms the pathogenesis of the disease can be divided into a number of different stages. Following an unknown trigger there is vascular dendritic cell activation which causes activation and polarisation of CD4 + T cells [16, 17]. The pro-inflammatory cytokines shift T-cell differentiation towards Th17 and Th1 cells [18]. The Th17 cells are reliant on Interleukin (IL)-6 and produce IL-17 (amongst other interleukins); this cluster predominants early in GCA and fluctuates with disease activity. Importantly this cluster are highly responsive to standard glucorticoid therapy [19]. Whereas IL-12 and IL-18 induce Th1cells that release interferon (IFN)-y are associated with chronic disease and more resistant to glucorticoids [18–20].

## Ageing processes and GCA

**Fig. 1:**
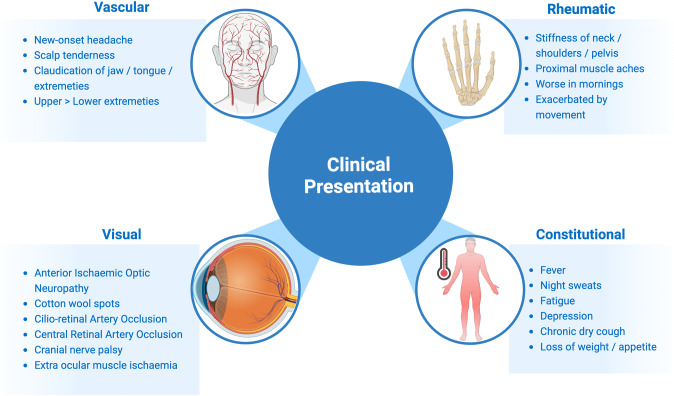
Schematic highlighting the clinical presentation features of GCA. These can be cateogorised into vascular symptoms, visual signs, rheumatology and constitutional symptoms.

A number of risk factors for the development of GCA have been identified such as history of vascular disease, smoking, low body mass index and early menopause [21–24], however ageing has been found to be the strongest of all risk factors [24] (Fig. 1). GCA almost exclusively affects individuals aged 50 or older [14]. Vascular ageing may play a central role in the initial immune activation in GCA. Ageing has been known to make blood vessels vulnerable to damage and inflammation, with coining of the term “inflammaging” and atherosclerosis being described as a “prototypical form” of vascular ageing [25–28]. Multiple pathways are believed to contribute to vascular ageing, including oxidative stress, mitochondrial dysfunction, chronic low-grade inflammation, cellular senescence, increased apoptosis, epigenetic alterations, genomic instability, and clonal haematopoiesis of indeterminate potential (CHIP) [27, 29].

Population studies have observed that ageing is associated with chronically higher circulating levels of pro-inflammatory cytokines and inflammatory markers, namely, IL-6, IL-18, IL-1ra, C-reactive protein (CRP), and fibrinogen [30]. It is unclear whether these inflammatory markers are a product of ageing alone, as many studies have associated their increased prevalence with the presence of cardiovascular risk factors, which are particularly ubiquitous in elderly populations [30, 31]. IL-6 has been associated with clonal hematopoiesis of indeterminate potential (CHIP), a pre-malignant state characterised by somatic mutations in hematologic precursor cells is another potential pathogenetic mechanism potentially implicated in development of GCA [32]. The incidence of CHIP correlates with age, and it is associated with increased levels of CRP and other classic systemic inflammatory markers [33, 34]. Preliminary works exploring a potential correlation between CHIP and the development of GCA seem to corroborate this association [35].

Another theory is the potential role of somatic variants (SV) in GCA, as the number of SVs increases with ageing. SVs are postzygotic, mutations acquired during mitosis or after exposure to endogenous (i.e. products of cellular metabolism, reactive oxygen, and nitrogen species) or exogenous factors (i.e. ultraviolet light or radiation, tobacco, and alcohol), eventually leading to mosaicisms. SVs can render immune system cells resistant to apoptosis or change their functional profile (i.e. leading to aberrant cytokine secretion), causing high-inflammatory, non-proliferative (i.e. non-neoplastic) immune disorders [36].

The link between GCA and atherosclerosis remains ambiguous. Atherosclerosis has overlapping pathophysiology with GCA as cytolytic, proteolytic and reactive oxygen species are deposited in arterial adventitia, causing chronic low-grade inflammation, angiogenesis and fibrosis, subsequently leading to arterial remodelling [37]. The remodelling process is also characterised by T-cell, macrophage and mast cell migration into the adventitia, causing collagen breakdown by Matrix metalloproteinase 9, compromising the previously immunoprivileged arterial wall [38]. Vascular remodelling may occur early in atherosclerotic disease, indeed a study on porcine coronary arteries in the context of a high cholesterol diet found adventitial vasa vasorum remodelling through neovascularization occurred prior to atheromatous plaque formation [39]. Due to the shared pathological processes, one might predict that the presence of GCA or atherosclerosis could precipitate or accelerate the development of the other, however others found that GCA incidence inversely correlates with cardiovascular risk factors (obesity, smoking, hyperglycaemia, hypercholesterolaemia), and co-existent findings of GCA and atherosclerosis are rare on temporal artery biopsies [28, 40, 41]. The underlying protective mechanism of atherosclerosis and GCA currently remains unclear, however hyperglycaemia has been speculated to impair T-cell function, suppressing the inflammatory response in GCA [28].

## Clinical presentation

**Fig. 2:**
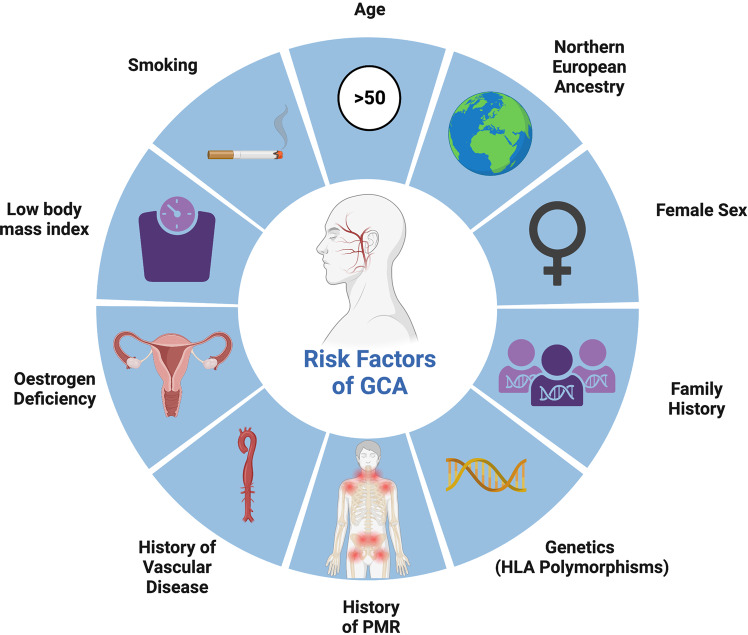
Risk factors for GCA.

GCA has heterogeneous clinical features due to the overlapping spectrum of the known clinical phenotypes: cranial GCA (C-GCA), large vessel GCA (LV-GCA), and polymyalgia rheumatica (PMR) (Fig. 2) [42]. The majority of people with cranial GCA will have symptoms of new onset headache, jaw claudication and cutaneous allodynia [8, 43, 44]. Nearly half of people with GCA have symptoms of PMR (Fig. 2) while up to one-fifth of people with PMR will be diagnosed with GCA [5, 42]. There may be large vessel involvement in cranial GCA, which may be asymptomatic and revealed by diagnostic imaging alone [45, 46]. Up to 50% of people with GCA will experience constitutional symptoms such as fever, weight loss, night sweats, loss of appetite, malaise, depression [16]. These may help narrow the differential diagnosis from an ocular cause or pain syndrome (such as migraine or cluster headache) to a systemic cause, however many systemic conditions have the potential to exhibit these symptoms (Table 1).

**Table 1:** Potential differential diagnoses for those suspected with GCA.

Diagnostic sieve	Potential differential diagnosis for those with suspected GCA
Ocular causes	Non-arteritic anterior ischaemic optic neuropathy
	Angle closure glaucoma
Regional causes	Carotid artery occlusive disease
	Intracranial pathology (such as pituitary apoplexy or cavernous sinus lesion)
	Migraine
	Cluster headache
	Trigeminal neuralgia
	TMJ dysfunction
	Dental pain
	Cervical spondylosis
Infections	Herpes zoster ophthalmicus
	Varicella zoster
	Otitis external
	Sinusitis
	Dental abscess
Systemic conditions	Multiple myeloma
	Amyloidosis
	Other vasculitides (such as granulomatosis polyangiitis)

The visual symptoms of GCA are well documented and include amaurosis fugax, double vision to devastating visual loss [47, 48]. The most common ocular manifestations include anterior ischaemic optic neuropathy [49], large peripapillary cotton wool spots [50, 51], arterial occlusions (cilio-retinal artery or central artery) [50, 52], oculomotor cranial nerve palsy [47], and posterior ischaemic optic neuropathy [50], with other rarer ocular syndromes having been reported (Table 2) [53–61]. Initiation of treatment may result in reversal of visual loss in the minority [62, 63].

**Table 2:** Ocular manifestations of Giant Cell Arteritis.

Presentation	Ocular manifestations of Giant Cell Arteritis
Very common	Anterior ischaemic optic neuropathy
Common	Cotton wool spots
	Cilio-retinal Artery Occlusion
	Central Retinal Artery Occlusion
	Cranial nerve palsy
	Extra ocular muscle ischaemia
Rare	Posterior ischaemic optic neuropathy
	Choroidal ischaemia
	Anterior segment ischaemia
	Anisocoria (ischaemic mydriasis, tonic pupils, Horner’s syndrome)
	Ocular ischaemic syndrome
	Homonymous hemianopia
Very rare	Peripheral Ulcerative Keratitis
	Scleritis
	Orbital pseudotumour

Ophthalmologists need to beware of the less common presentations of GCA, as they may be asked to examine patients who are suspected of having GCA. For example, symptoms of LV-GCA include intermittent limb claudication or absent pulses according to the vessels affected and chest or back pain if there is aortic involvement [64]. GCA can present without any symptoms of cranial or large vessel involvement, with inflammation or fever of unknown origin (IFUO), anorexia, weight loss and anaemia being the only evidence of an active disease process. Patients with constitutional GCA are at risk of significant diagnostic delay due to the large differential diagnosis of IFUO [65, 66].

## Confirming a diagnosis of GCA

GCA diagnosis is made on a clinical basis, in conjunction with laboratory, temporal artery biopsy (TAB), or vascular imaging evidence, as the clinical findings can help improve pre-test probability. A key challenge in urgent clinical practice is the heterogenic presentation of GCA and the wide differential of possible diagnoses [44, 67] (Table 1). Modern publications have suggested regression, neural networks, machine learning models, or clinical scoring systems however all of these rely on complete clinical information about the individual patient. Most of these tools are yet to be validated in larger, unbiased and well-proportioned datasets [68, 69].

There are currently no diagnostic criteria for GCA, however classification criteria such as the American College of Rheumatology (ACR) are often used inappropriately for the purpose of diagnosis [70]. Such classification criteria are for research purposes and exclude symptoms that are commonly found across multiple disease entities, focussing predominantly on signs and symptoms found solely in certain disease entities and not others. Key developments in the field of GCA have now been incorporated into the 2022 ACR/European League Against Rheumatism (EULAR) classification criteria for GCA which include the advancements in ultrasound and PET imaging (Table 3) [71]. The 2022 ACR/EULAR criteria have been validated for research purposes in the Diagnostic and Classification Criteria for Vasculitis (DCVAS) data set. This was across the disease spectrum (biopsy proven-GCA versus L-GCA) and in different populations of North America and Europe [71]. The previous 1990 ACR had good sensitivity and specificity of 93.5% and 91.2%, respectively, when differentiating C-GCA from other types of vasculitis, but performed poorly when used for diagnostic purposes [70]. Indeed, a retrospective case series has shown that 25.7% of patients with a positive TAB did not meet the 1990 ACR criteria, highlighting the that these criteria are not intended for diagnostic purposes [72].

**Table 3:** Adapted from the 2022 American College of Rheumatology/ European Union League Against Rheumatism Classification Criteria for GCA.

Mandatory requirement for all	Age at time of diagnosis ≥ 50 years
Score	Feature
+5	Positive temporal artery biopsy or halo sign on temporal artery ultrasound
+3	Maximum ESR ≥ 50 mm/hour or maximum CRP ≥ 10 mg/Litre prior to the initiation of treatment
+3	Sudden visual loss
+2	New temporal headache
+2	Jaw or tongue claudication
+2	Scalp tenderness
+2	Morning stiffness in the shoulders/neck
+2	Abnormal examination of the temporal artery
+2	Bilateral axillary involvement
+2	FDG-PET activity throughout the aorta

Total score ≥ of 6, with alternate diagnoses excluded, is needed for a classification of GCA.

*C-RP* C-reactive protein, *ESR* erythrocyte sedimentation rate, *FDG-PET* fluorodeoxyglucose (FDG)-positron emission tomography (PET).

## Laboratory markers

There are currently no specific routine serological markers to definitively diagnose GCA. Commonly performed blood tests to identify an inflammatory state include CRP; erythrocyte sedimentation rate (ESR) or plasma viscosity (PV); and platelet count. None are specific, however used in combination they may provide more diagnostic certainty in combination with the clinical findings, vascular imaging, or TAB [15]. The difficulty is that most inflammatory and infective aetiologies share a similar biochemical profile (Table 1). In clinical practice certain tests may not be available in the local laboratory. For example, some labs have chosen not to perform ESR and instead offer plasma viscosity as it is not affected by haematocrit variations (e.g. anaemia or polycythaemia) nor affected by a delay in analysis. The challenge here is familiarity as most publications have evaluated ESR and not PV for the diagnosis of GCA. Another diagnostic dilemma is that the ESR and CRP values have been documented as normal in people with GCA [73–75]. A thrombocytosis >400,000/μL has shown to be beneficial at predicting a positive biopsy result [76]. Hence, the combination of ESR, CRP, and platelet count has been recommended to provide most useful biochemical information to predict GCA probability [15].

## Temporal artery examination

Clinical examination of the temporal arteries by palpation is a critical assessment. Signs of abnormality include absent or diminished pulses, tenderness or a hard “cord-like” structure [16, 67]. Temporal artery biopsy (TAB) has long been held as the “gold-standard” investigation for GCA due to its ability to provide a histopathological tissue diagnosis, with reported specificities as high as 100% [77]. To estimate the sensitivity of unilateral TAB for the diagnosis of GCA a meta-analysis based on a large sample size found the sensitivity to be 77% [78]. There are a number of reasons for the reduced sensitivity including inadequate sample length, incorrect tissue sampled and the initiation of steroids prior to biopsy [5, 8, 16]. Another widely known factor limiting the sensitivity of TAB is the presence of skip lesions in GCA [79]. Skip lesions are estimated to be present in 8–26% of cases and therefore risk false negative results if biopsies are sampled from spared segments of arteries [80, 81]. To improve the sensitivity of detecting GCA some clinicians have advocated bilateral simultaneous TABs. However there has been a wide range of discordance rates between 3% to 45% found in people undergoing bilateral simultaneous TABs [82]. One option, where frozen section is available, is to perform a unilateral biopsy and if this is positive on frozen section it avoids the contralateral biopsy [82]. The practice of performing bilateral simultaneous TAB versus unilateral TAB is known to be different worldwide [83]. Ophthalmologists can be asked to perform TAB in a person without cranial symptoms and a clinically relevant finding is that a tertiary cohort study found that only 52% of patients with LV-GCA had positive TAB results, making the investigation relatively biased towards C-GCA [84].

There has been relatively little comment in the literature regarding the criteria for which histopathologists regard as positive for GCA and their agreement, until the advent of ultrasound [77]. Indeed, there are no internationally accepted criteria for a positive temporal artery biopsy [71]. Importantly when balancing the validity of TAB histopathology assessment versus the use of US to diagnose GCA one study reporting moderate agreement between 12 trained sonographers (κ= 0.61) assessing 20 ultrasound videos and 14 pathologists (κ = 0.62) assessing 30 TAB biopsy images [77].

Histopathological features of GCA include presence of giant cells, transmural evidence of mononuclear or granulomatous medial inflammation, internal elastic lamina fragmentation, necrosis, arterial mural thickening and/or intraluminal thrombosis. The concept of perivascular adventitial inflammation alone representing a spectrum of GCA pathology has sparked much debate in recent years [83]. Perivascular inflammation is restricted to the adventitia and periadventitial structures, and encompasses small vessel vasculitis, vasa vasorum vasculitis and inflammation limited to adventitia. It has been estimated to be present in 5–9% of positive TAB biopsies and has fair specificity ranging from 81.4 to 88.1% for GCA [82–87]. However, others have reported poor positive predictive values of perivascular inflammation, associating its occurrence with anomalies of ageing, systemic inflammation, malignancy and PMR phenotypes rather than relation to GCA directly [79, 88–92].

## Temporal and axillary artery ultrasound

Over recent years, the availability and refinement of imaging services have improved in healthcare settings, with their rapid incorporation into diagnostic and interventional modalities for a multitude of pathologies. The same holds true for GCA; the EULAR currently recommend the use of temporal and axillary artery ultrasound (US) to confirm the diagnosis of new GCA cases, given the low invasiveness, rapid result availability, and comprehensive inflamed vessel visualisation of the imaging modality [45]. Temporal artery ultrasound has been found to be a cost-effective alternative to TAB in reducing false negatives, with US providing a £485 benefit per patient [77]. In situations where US is not available or has limited utility (e.g. thoracic aorta assessment), EULAR recommends the use of cross-sectional imaging such as MRI, CT and PET to aid GCA diagnosis in the first instance [45].

Four pathological signs are found by US in GCA: halo sign, compression sign, stenosis, and vessel occlusion [93]. When viewed using ultrasound, inflammatory tissue is hypoechoic, allowing a skilled sonographer to detect halo sign (hypoechoic artery wall thickening), and compression sign (hypoechoic vessel wall infiltrate in the presence of arterial lumen occlusion), which were initially reported to have similar sensitivity and specificity to TAB of 79% and 100%, respectively [94]. However, a recent meta-analysis comparing three GCA US signs (halo sign and temporal artery compression/stenosis) with temporal biopsy reported lower sensitivity and specificity of 68% and 81% respectively [95]. Adequate and structured training is an important consideration to improve the reliability of US in GCA diagnosis [96].

A single centre study has used halo sign thickness to develop Halo scores, which were associated with markers of systemic inflammation such as CRP, platelet count and haemoglobin, but not ESR [97]. Halo scores of ≥2 have been associated with ocular ischaemic events including anterior and posterior ischaemic optic neuropathy and the presence of a relative afferent pupillary defect (OR 12.00, *p* = 0.022), with scores of ≥10 conveying a specificity of 95% for GCA diagnosis, inferring their potential utility in diagnosis identifying those at risk of poor visual outcomes [97].

Ultrasonography holds great promise for diagnosing forms of GCA other than cranial. Temporal arteries can be spared in 40% of patients with LV-GCA, risking misdiagnosis when relied upon in isolation for diagnosis [98]. LV-GCA has been associated with delayed diagnosis and worse clinical outcome, with many requiring a higher cumulative glucocorticoid dose, and are at higher risk of relapse and aneurysm development [78, 99, 100]. Axillary artery involvement has been noted up to 98% in confirmed LV-GCA cases [99].

There is currently debate regarding the sensitivity of US in GCA diagnosis after starting glucocorticoid treatment. Some have found the sensitivity to decrease [92], others have found the majority of temporal and minimal numbers of axillary artery haloes take weeks to disappear [97, 101–104]. Conversely, TAB histological results remain positive for a prolonged period of time, with biopsies taken from 3 to 4 cm TAB segments showing persistent abnormal cell infiltrates in 70–75% of patients in the first 6 months, and 44% of patients within 9–12 months of starting corticosteroid therapy, making TAB the preferred investigation of choice in cases with significant delay in referral times [105]. Despite the evidence of persistent pathological features whilst receiving glucocorticoid therapy, clinicians are currently recommended to scan as early as feasible due to variability in patient response to glucocorticoid treatment [91]. There is a sizeable divergence of opinions on which test should be considered as the “gold standard” to diagnose GCA [83]. Many clinicians scrutinise the value TAB and US as separate entities, however the paradigm of one-test-to-diagnose-them-all might be considered a myopic standpoint. Appreciation for the individual test’s strengths and weaknesses, in combination with comprehensive history taking and examinations are fundamental in the work up of GCA [7, 106–109].

## Rapid access GCA pathways

Early glucocorticoid treatment is associated with improved ophthalmological outcomes, with diagnostic delays risking ophthalmic ischaemic events [110]. Cranial presentations accrue a mean diagnostic delay of 7.7 weeks, with non-cranial presentations receiving longer delays of 17.6 weeks, risking permanent visual loss if glucocorticoids have not been initiated, and difficulty detecting diagnostic pathological features if they have [111].

Fast-track GCA referral pathways utilising rapid access to specialist assessment and imaging modalities within one working day were first recommended internationally for GCA diagnosis by EULAR in 2018 but have been employed by institutions since 1997 [45, 93]. The introduction of fast-track referral services have been shown to decrease rates of permanent vision loss and reduced diagnostic delay [112–114]. Fast track services have been shown to reduce the need for TAB by up to 93%, with the majority of TAB being performed due to inconclusive US findings [113]. Such pathways have undergone refinement since their inception. A pre-test probability score has been developed which allows risk-categorisation and algorithmic processing of referrals and has shown promise in its ability to identify non-GCA referrals, reporting a sensitivity of 100% and specificity of 48.2% in patients scored as “low risk” (≤9 points) [115]. Validation of this score is currently in early stages, however it could prove a helpful tool for referrers or diagnosticians if further studies corroborate its use. A number of UK National Health Service Trusts have fast track services, and further work is required to make these the standard of care.

## Management of GCA

All treatment recommendations are well considered in the recent guidelines such as the EULAR and British Society of Rheumatology guidelines [4, 5]. High dose glucocorticoids should be started once GCA is suspected [4, 5, 8]. In a randomised control trial use of intravenous methylprednisolone versus placebo in the first 3 days of treatment in combination with oral prednisolone 40 mg/day observed faster glucocorticoid taper, reduced cumulative glucocorticoid dosing and fewer relapses in the methylprednisolone arm, as compared to placebo [116]. It is worthy of note that those with visual loss were excluded from this trial [116]. Guideline groups have debated the use of intravenous glucocorticoids for initiation of therapy but there is a lack of good evidence to conclusively recommend their mandated used [4, 5]. Relative contraindications to intravenous glucocorticoid therapy may include uncontrolled hyperglycaemia, diabetes mellitus, osteoporosis and facture, and other medical conditions that the clinician would need to weigh up the relevance such as a recent history of pancreatitis, uncontrolled mental health disorders, or congestive heart failure [5, 117, 118].

The burden of side effects, and their management, from long-term glucocorticoids in GCA are well known [119–121]. Gradual and controlled glucocorticoid reduction is imperative, in order to balance the risk of flare or relapse versus the risk of metabolic side effects related to their use [119–121]. However, there is a mismatch between recommended steroid tapering regimens and real world data, where cumulative doses of glucocorticoids have been three times higher than recommended [122, 123].

A number of different conventional synthetic and biologic disease-modifying anti-rheumatic drugs (DMARD) have been trialled in GCA [67, 124]. Low-dose methotrexate demonstrated a modest reduction in relapse and cumulative glucocorticoid dose at meta-analysis [125], and is used routinely in clinical practice in the UK and Europe [4, 5]. Leflunomide, another conventional synthetic DMARD suppresses the production of pro-inflammatory cytokines through the activation of dendritic cells and modifies the action of the T-cell response in GCA [126]. A number of studies support its use, but as of yet there is no randomised controlled evidence for its directed use [127–130].

There remains no conclusive evidence from controlled trials to determine the safety and efficacy of low-dose aspirin as an adjunctive treatment in GCA. A portion of people with GCA will be on aspirin at time of the diagnosis, and aspirin does not need to be discontinued [5]. There is not good enough evidence to consider the use of low-dose aspirin as an adjunctive treatment for GCA and clinicians must recognise the established haemorrhagic risks associated with aspirin, especially in the context of concurrent treatment with glucocorticoids [131].

Targeted treatment with subcutaneous Tocilizumab (TCZ) has shown significant glucocorticoid-sparing effects in new-onset and relapsing patients with GCA [132–134]. TCZ is a monoclonal antibody directed against the IL-6 receptor that inhibits signalling by the pro-inflammatory cytokine IL-6 [135]. In the landmark study Giant-Cell Arteritis Actemra (GiACTA), 249 patients with new onset GCA or refractory disease were enrolled and randomised to one of four arms: weekly (TCZ QW) or fortnightly (TCZ 2QW) dosing of TCZ with a 26-week prednisone taper or placebo plus a 26-week or 52-week prednisone taper. At 52 weeks, patients in the TCZ groups were significantly more likely to have achieved sustained remission as compared with both the 26-week and 52-week glucocorticoid taper groups, and at just over half the cumulative glucocorticoid dose [133]. The data from the GiACTA at 52 weeks showed that the outcomes of patients with new-onset disease at baseline who were randomly assigned to TCZ Q2W did not clearly differ from the outcomes of patients who received TCZ QW, and TCZ QW was more effective than TCZ Q2W for these outcomes in patients with relapsing disease at baseline [133]. At 3 years following the GiACTA trial and open label follow-up, treatment once weekly (TCZ QW) overall delayed time to flare and reduced glucocorticoid exposure in patients with both new-onset and relapsing GCA as compared to those treated TCZ 2QW [136, 137].

Replicating the GiACTA trial results in the real world may be challenging as both placebo arms had a significantly faster glucocorticoid taper than used in routine clinical practice, and one third of patients had a diagnosis of GCA based on large vessel imaging, not ultrasound [133]. It is important also to note that use of intravenous glucocorticoid therapy was a specific exclusion criterion in GiACTA, which would have negatively biased against enrolling those with visual loss. Delivery of TCZ in the UK is devolved to the four nations [138, 139], and new data from Scotland possibly suggests under-utilisation of TCZ in terms of those with relapsing disease or those with high-risk comorbidities [140]. Conway et al. [141] thoughtfully discuss the Scottish findings in the context of prescribing confidence of a newly licensed biological agent and on the backdrop of the COVID-19 pandemic which changed many GCA pathways and practices [142, 143].

## Visual outcomes in GCA

Visual outcomes in those with visual loss secondary to GCA is poor, with little chance of recovery [62, 144]. While intravenous methylprednisolone has been used since the 1990s, there is not good enough evidence to determine if it actually prevents visual loss. One early case series concluded that use of intravenous methylprednisolone was no better than using high dose oral prednisone [145]. In another study where patients were treated with a standard protocol of 1 g of intravenous methylprednisolone daily for 3 days followed by oral prednisone 60 or 80 mg (depending on patient weight), visual deterioration was noted in 27% of eyes, with the greatest risk of deterioration observed within the first 6 days [62].

An enduring clinical concern in immediate or long-term follow-up is what is the ongoing risk of visual loss in a person with GCA and concurrent treatment, and when treatment has ceased. A recent longitudinal study found the incidence of permanent visual loss to be around 2.2%, which was corroborated by the pooled incidence in the literature of 2.8% [146]. Those at risk are people with an established ischaemic event (such as contralateral visual loss from anterior ischaemic optic neuropathy), and it appears that the risk may be higher at initiation of treatment but can also occur when glucocorticoids are tapered. More data is required to understand the beneficial impact of targeted therapies, such as TCZ on rates of visual complications [63, 133, 147].

## Conclusion

In this global health care environment different attitudes, variable access to medicines and what is recommended by country specific guidelines informs the clinician and indeed the literature moving forward [4–6]. The challenge for Ophthalmologists who routinely investigate and manage GCA, is whether they have optimised their treatment for each individual patient whether it be early in the disease or further down the line. The importance of collaborative working with Rheumatology specialists, who have in depth experience of second line therapies, cannot be over-estimated [5, 67]. Targeted treatment remains an individualised approach that is required to balance the burden of treatment against its effectiveness at reducing relapses and inducing sustained remission [148]. There remain many unanswered questions, particularly pertaining to visual loss in GCA and whether this can be minimised or ideally even prevented.
